# Diversity and Resistance Profiles of Human Non-typhoidal *Salmonella* spp. in Greece, 2003–2020

**DOI:** 10.3390/antibiotics10080983

**Published:** 2021-08-15

**Authors:** Kassiani Mellou, Mary Gkova, Emily Panagiotidou, Myrsini Tzani, Theologia Sideroglou, Georgia Mandilara

**Affiliations:** 1 National Public Health Organization, 15123 Maroussi, Greece; k.mellou@eody.gov.gr (K.M.); biostat1@eody.gov.gr (M.G.); t.sideroglou@eody.gov.gr (T.S.); 2 National Reference Centre for *Salmonella*, School of Public Health, University of West Attica, 11521 Athens, Greece; empanagiotidou@uniwa.gr; 3 General Veterinary Directorate, Hellenic Ministry of Rural Development and Food, 10176 Athens, Greece; pavloszalimidis@yahoo.com

**Keywords:** *Salmonella* spp., Greece, serotype, antimicrobial resistance, laboratory surveillance

## Abstract

*Salmonella* spp. is one of the most common foodborne pathogens in humans. Here, we summarize the laboratory surveillance data of human non-typhoidal salmonellosis in Greece for 2003–2020. The total number of samples declined over the study period (*p* < 0.001). Of the 193 identified serotypes, *S*. Enteritidis was the most common (52.8%), followed by *S*. Typhimurium (11.5%), monophasic *S*. Typhimurium 1,4,[5],12:i:- (4.4%), *S*. Bovismorbificans (3.4%) and *S*. Oranienburg (2.4%). The isolation rate of *S*. Enteritidis declined (*p* < 0.001), followed by an increase of the less common serotypes. Monophasic *S*. Typhimurium has been among the five most frequently identified serotypes every year since it was first identified in 2007. Overall, *Salmonella* isolates were resistant to penicillins (11%); aminoglycosides (15%); tetracyclines (12%); miscellaneous agents (sulphonamides, trimethoprim, chloramphenicol and streptomycin) (12%) and third-generation cephalosporins (2%). No isolate was resistant to carbapenems. In total, 2070 isolates (24%) were resistant to one or two antimicrobial classes and 903 (10%) to three and more. Out of the 1166 isolates resistant to fluoroquinolones (13%), 845 (72%) were *S*. Enteritidis. *S*. Enteritidis was also the most frequently identified serotype with a resistance to third-generation cephalosporins (37%, 62/166), followed by *S*. Typhimurium (12%, 20/166). MDR was most frequently identified for *S*. Typhimurium and its monophasic variant (resistant phenotype of ampicillin, streptomycin, tetracycline and sulphamethoxazole with or without chloramphenicol or trimethoprim).

## 1. Introduction

Human non-typhoidal salmonellosis persists in industrialized countries, despite the advanced personal, domestic and community sanitation and hygiene conditions and monitoring of food processing. The main mode of transmission is via the consumption of food products usually of animal origin, most commonly eggs and poultry but also undercooked meat, poor meat handling, unpasteurized dairy products, seafood, fresh produce and fruits [1,2]. Non-typhoidal *Salmonella* spp. (NTS) cause infections that may have a variety of clinical manifestations; gastroenteritis is the most common of them, and it is usually self-limiting. A less frequent but more severe manifestation is bacteremia. NTS serotypes represent some of the most common causes of foodborne illnesses worldwide and, therefore, have a major economic and public health impact [3]. In the European Union (EU), salmonellosis is the second-most reported gastrointestinal infection in humans after campylobacteriosis and a major cause of foodborne outbreaks; in 2019, 87,923 *Salmonella* cases were reported, 16,628 hospitalized cases and 140 deaths. The EU notification rate for 2019 was 20.0 cases per 100,000 population [4].

NTS are responsible for the highest annual burden and the largest number of deaths both globally and in the European Region among foodborne hazards. Globally, NTS was estimated to cause approximately 78 million cases of illness, 59,000 deaths and four million (disability-adjusted life years) DALYs per year [5]. In the European Region, NTS are estimated to annually cause 107,000 DALYs and 1854 deaths [6].

The laboratory surveillance of *Salmonella* spp. is important to (a) monitor the temporal distribution of different strains, (b) evaluate the implemented control programs targeting salmonellosis in the country and (c) identify the emerging serotypes and changes in the antimicrobial resistance profiles, aiming at guiding public health strategies regarding salmonellosis. In this paper, we summarize the available data from laboratory surveillance of non-typhoidal human salmonellosis in Greece for the period 2003–2020.

## 2. Results

### 2.1. Serotyping Results

The National Reference Centre for Salmonella (NRCS) received 14,140 clinical isolates through the study period, of which 10,513 were fully serotyped. The exclusion of (para)typhoidal isolates (*n* = 157) yielded a total of 10,356. Of them, 10,065 isolates belonged to *Salmonella enterica* subspecies *enterica* and 291 to other subspecies: *S. enterica salamae* (*n* = 252) and *S. enterica diarizonae* (*n* = 39). A time–trend analysis showed a statistically significant decline of the number of non-typhoidal isolates from 2003 to 2020 (*p* < 0.001) and the seasonality of isolation rate with an increased number of isolates during the summer months, with peaks in August and September.

Among the 10,065 *Salmonella enterica enterica* subspecies isolates, a total of 193 serotypes were identified. The median number of different serotypes identified per year was 55 (range: 26–64). There was no trend in the number of different serotypes identified per year.

*Salmonella enterica enterica* serotype Enteritidis (*S*. Enteritidis) was the most frequently identified serotype, representing almost 53% of the total number of isolates. *Salmonella enterica enterica* serotype Typhimurium (*S*. Typhimurium) accounted for 12%, followed by its monophasic variant *Salmonella enterica* subsp. *enterica* with antigenic type 1,4,[5],12:i:- (monophasic *S*. Typhimurium) with 4%; *Salmonella enterica enterica* serotype Bovismorbificans (*S*. Bovismorbificans) and *Salmonella enterica enterica* serotype Oranienburg (*S*. Oranienburg) are included in the five more frequently identified serotypes, accounting for 3.4% and 2.4% of the isolates, respectively). *S. enterica* subsp. *salamae* with the antigenic type 1,4,[5],12,[27]:b:- is a serotype circulating constantly in Greece, although the annual number of isolates is low (Table 1).

**Figure 1:**
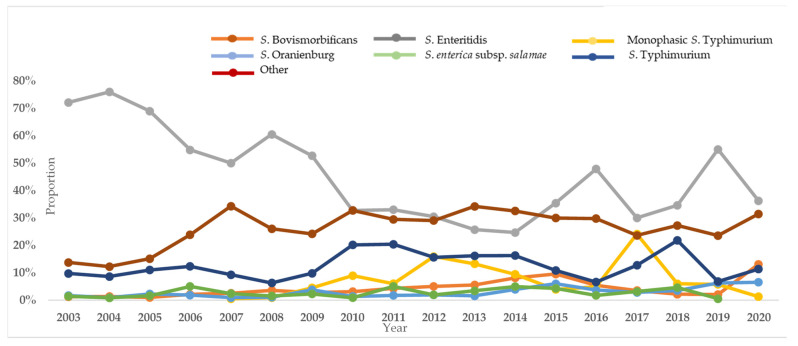
The proportion (%) of *S*. Enteritidis, *S*. Typhimurium, Monophasic *S*. Typhimurium, *S*. Bovismorbificans, *S*. Oranienburg, *S. enterica*. subsp. *salamae* and of all other serotypes by year, Greece, 2003–2020.

The annual proportion of *S*. Enteritidis, *S*. Typhimurium, monophasic *S*. Typhimurium, *S*. Bovismorbificans, *S*. Oranienburg, *S. enterica*. subsp. *salamae* and of all the other serotypes over the total number of isolates is depicted in Figure 1. For the years 2003–2005, the average proportion of *S*. Enteritidis among the serotyped isolates was 72%, for the next four years, 2006–2009, the average dropped (55%) and, for the other 11 years, a further decrease was recorded (up to 35%). The decrease of *S*. Enteritidis isolation was statistically significant (*p* < 0.001). This decrease was followed by the increase of the proportion of the “other serotypes”; for the years 2003–2005, the average frequency of the “other serotypes” was 14%, and for the other 15 years, it increased up to 30%. The *S*. Typhimurium isolation rate did not present a significant trend during the study period; however, its monophasic variant was identified in Greece for the first time in 2007 and, since then, has been among the five most frequently identified serotypes. *S*. Oranienburg and *S*. Bovismorbificans circulate in Greece in more or less stable numbers.

### 2.2. Age, Sex and Type of Sample

**Figure 2:**
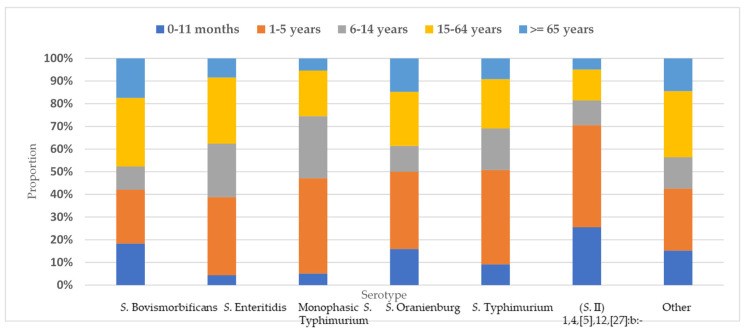
The distribution of *Salmonella* spp. per serotype by age group, Greece, 2003–2020.

Data on the ages of the persons whose specimens yielded isolates were available for 7036 isolates (Table 2). The highest detection rate was observed in the age group of 1–5 years (34%), followed by the age group of 15–64 years (28%). The age groups of 0–11 months and over 65 years accounted for 8.6% and 10.1% of the cases, respectively. When the age groups of 0–11 months and 1–5 years were analyzed together, the detection rate for all the serotypes was between 40% and 50%, with the exception of *Salmonella enterica* subsp. *salamae* (*S*.II) antigenic type 1,4,[5],12,[27]:b:-, for which almost 70% of the isolates were recovered in children <5 years old (Figure 2).

Data on the sex of the patients (9491 isolates) revealed an equal distribution in males and females (51.5% and 48.5%, respectively).

Information on the type of specimen where *Salmonella* spp. were isolated was available for 9687 isolates (Table 3). The majority was isolated from stool samples (almost 94%) and 4.4% (*n* = 429) from blood. Of the 429 isolates from blood, 204 (47.5%) were *S*. Enteritidis, 56 (13%) *S*. Typhimurium and 28 (6.5%) *S*. Oranienburg. *S*. Oranienburg presented the highest isolation rate from the blood samples (12.5%, 28/224).

### 2.3. Antimicrobial resistance profile (AMR)

Data on the AMR were available for 8703 *Salmonella* spp. isolates (Table 4). During the studied period, the *Salmonella* isolates were resistant to penicillins (11%); aminoglycosides (15%); tetracyclines (12%) and miscellaneous agents (sulphonamides, trimethoprim, chloramphenicol and streptomycin) (12%). The resistance was mainly attributed to *S*. Typhimurium and to the monophasic variant of *S*. Typhimurium with the multi-resistant phenotype of ASSuT (ampicillin, sulphamethoxazole, streptomycin and tetracycline). The occurrence of resistance to the highest priority critically important antimicrobials and, thus, to the antimicrobials currently used in medicine (mostly also used in veterinary medicine) [7] was 13% to fluoroquinolones and 2% to third-generation cephalosporins. From the 1136 isolates resistant to fluoroquinolones for the period 2003–2016, all of these were resistant to nalidixic acid and seven to ciprofloxacin. From 2017, when ciprofloxacin was replaced by pefloxacin, to 2020, 25 isolates were resistant to both nalidixic acid and pefloxacin and five to pefloxacin only. Overall, 72% (845/1166) of the isolates resistant to fluoroquinolones were *S*. Enteritidis. *S*. Enteritidis was also the most frequently identified serotype, with a resistance to third-generation cephalosporins (37%, 62/166), followed by *S*. Typhimurium (12%, 20/166) and many other serotypes represented by one to five isolates (e.g., *S*. Kentucky, *S*. Virchow, *S*. Kottbus and *S*. Hadar).

**Figure 3:**
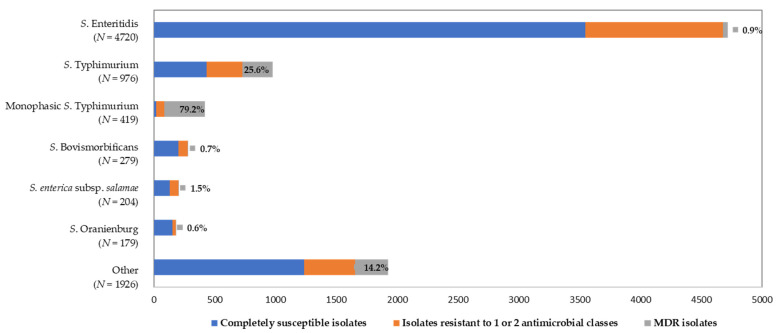
The proportions of the multidrug-resistant (MDR) isolates, isolates resistant to 1 and/or 2 antimicrobial classes and completely susceptible *Salmonella* spp. isolates from humans in Greece, 2003–2020.

In total, 2070 isolates (24%) were resistant to one or two antimicrobial classes and 903 (10%) to three or more. *S*. Enteritidis, which accounts for 53% of the serotyped isolates, did not exhibit a high antimicrobial multi-resistance (less than 1%). A MDR was most frequently identified for *S*. Typhimurium and its monophasic variant (monophasic *S*. Typhimurium 1,4,[5],12:i:-) isolates with the resistant phenotypes of ampicillin, streptomycin, tetracycline and sulphamethoxazole with or without chloramphenicol or trimethoprim (Figure 3).

## 3. Discussion

In the present study, we summarize the laboratory data of non-typhoidal *Salmonella* isolates in Greece from 2003 to 2020. It is the first time that the assembled data from Greece is presented for such a long period of time. The number of clinical samples received by the NRCS from 2003 to 2020 decreased, which is a finding compatible with the data from the mandatory notification system that clinical cases are recorded in [8]. According to the Mandatory Notification System of the Hellenic National Public Health Organization (NPHO), during 2004–2019, the mean annual notification rate of salmonellosis was 5.9 cases per 100,000 population, and a statistically significant decreasing trend of the salmonellosis notification rate was observed during this period (*p* < 0.001) [8]. The decrease could be attributed to a decrease in the overall salmonellosis morbidity in the population. However, during the studied period, several social–economical and public health issues that took place, such as the rearrangement of the Hellenic public health system and the shutdown of several public hospitals and the Economic Adjustment Programme for Greece, and the COVID-19 pandemic may have also affected the number of clinical samples received by the NRCS. A previous study [9] estimated the actual underreporting for salmonellosis in Greece as 48%. The enhancement of the national surveillance system is of great public health importance in order to be robust and provide a wealth of epidemiological data to support decision-making.

*Salmonella enterica enterica* serotype Enteritidis is the most frequent serotype identified in Greece; however, it has presented a declining trend since 2005. The decrease was to be expected, as the National *Salmonella* Control Programmes (NSCPs) in poultry populations, which targeted mainly *S*. Enteritidis and *S*. Typhimurium, according to Regulation (EC) No 2160/2003, were implemented in Greece in 2008. According to the European Food Safety Authority (EFSA), eggs and poultry meat are the main sources of transmission for *S*. Enteritidis [4,10]. The positive impact of the implementation of control measures against *S*. Enteritidis has been documented in Greece [11], as well as in the other EU countries [4,10,12,13,14,15].

The increased isolation of other serotypes can possibly be attributed to the decrease of S. Enteritidis that may have left a niche for other serotypes to fill [16].

*Salmonella enterica enterica* serotype Typhimurium presented slight fluctuations during the studied period, but it was steadily the second-most common serovar. The same has been observed in the other EU countries; according to the EU annual summary reports on zoonoses [4,10,12,13,14], *S*. Typhimurium was the second-most frequently reported serotype accounting for, on average, 18% of serotyped isolates from 2004 to 2019. The monophasic variant of *S*. Typhimurium (1,4,[5],12:i:-) emerged in Greece in 2007 and, since then, has been among the five-most common serotypes, as it has been described in previous studies [17,18]. In the EU annual reports on zoonotic agents, monophasic *S*. Typhimurium was recorded for the first time in 2009. In the following years, the countries were harmonized with the method for the confirmation of the absence of the second phase of the flagellar antigen [19], which is now the third-most common serotype in European countries [4]. Both *S*. Typhimurium and its monophasic variant (1,4,[5],12:i:-) are mostly associated with pig (meat) (42% and 72%, respectively) and its widespread distribution in pork production [20]; since no national control program for *Salmonella* in pigs is in place in Greece, the presence of these serotypes is expected to be rather stable. Other serotypes frequently identified in Greece are *S*. Bovismorbificans, *S*. Oranienburg and *S*. Kottbus, which are not recorded regularly in EU countries (less than 0.6%) and, according to the literature, are associated with various food products (e.g., sesame seeds, ham, fruits, eggs, chocolate and vegetables) [21,22,23,24].

In total, the highest number of *Salmonella* isolates was recovered from young children <5 years old (42.5%). These data are consistent with studies from other parts of the EU [25]. Global reports also suggest that the highest risk for non-typhoidal salmonellosis is in children [6,26]. Nonfood-related risk factors may be of particular significance in infections in infants and very young children due to poor personal and home hygiene [26]. Children’s immature, innate and adaptive immune system should also be considered alongside the risks of salmonellosis [27]. Parents will apparently seek medical attention in the case of gastroenteritis symptoms of their child; hence, the isolation of the suspected pathogen from fecal or blood specimens is expected. It is worth noting that *Salmonella enterica* subsp. *salamae* with antigenic type 1,4,[5],12,[27]:b:- was recovered in very high percentages from children <5 years old. This finding requires further study for investigating the possibility of a specific food vehicle, since there is no relevant information in the literature.

There is a seasonal distribution of *Salmonella* recovery by month of isolation in the summer months, with peaks in August and September. Summer in Greece is characterized by high temperatures (31–36 °C), which favor the rapid multiplication of pathogens in any food matrices, a fact that is enhanced in cases of poor food handling and storage. Since Greece has a well-established tourism industry, the enhanced surveillance and prevention of foodborne diseases, including salmonellosis, is of great importance.

The majority of the *Salmonella* isolates of all serotypes were recovered from the stool samples. However, *S*. Oranienburg was recovered from the blood in a high percentage, indicating a more virulent potential. *Salmonella* bacteremia takes place when bacteria enter the bloodstream after invading the intestinal barrier. Almost all *Salmonella* serotypes can cause bacteremia [28]. Even though *Salmonella* serotypes are genetically closely related, they significantly differ in their pathogenic potential. According to a previous study, *S*. Oranienburg is one of the four *Salmonella* serotypes (Javiana, Montevideo, Oranienburg and Mississippi) that, among 21 NTS serotypes causing the majority of foodborne salmonellosis cases in the United States, encodes the *Salmonella* cytolethal distending toxin (S-CDT), an important virulence factor enabling them, more likely, to cause invasive disease [29,30].

Recently, Parisi et al. [31] suggested that infections caused by MDR *Salmonella* spp. strains have more serious health outcomes compared with pan-susceptible strains. In high-income countries, where person-to-person *Salmonella* infection is rare, several studies have provided evidence for a direct link between antimicrobial use in animals and the emergence of antimicrobial-resistant *Salmonella* infections in human [32,33]. Therefore, the surveillance of *Salmonella* spp. antimicrobial resistance (AMR) tracks the resistance trends in a microbial population, permits the early detection of resistant strains of public health importance, may correlate specific resistance profiles with a specific serotype and, hence, can enable timely intervention action plans. Moreover, it supports the prompt notification and investigation of outbreaks, mainly “open” diffused ones. AMR *Salmonella* surveillance data should also be taken into consideration in terms of clinical guidance when antimicrobial therapy is needed.

Although *S*. Enteritidis is the most frequently identified *Salmonella* serovar in humans in Greece, it is rarely resistant, with the exception of a resistance to fluoroquinolones; it rarely presents multidrug resistance. This fact suggests that the virulence properties of the respective isolates are more important than their resistance properties, as previously suggested [34]. According to the results concerning the AMR of human *Salmonella* spp. isolates in Greece, it seems that *S*. Typhimurium and its monophasic variant have affected the total resistance levels to ampicillin, streptomycin, sulphonamides and tetracycline, trimethoprim and chloramphenicol. Fortunately, a resistance to carbapenems was not observed, and the resistance to third-generation cephalosporins was very low, dispersed in many different serovars. In the EU countries in 2019, the resistance of human *Salmonella* spp. isolates to ampicillin, sulfonamides and tetracyclines was observed at, overall, high levels, mostly exhibited by *S*. Typhimuriun and its monophasic variant, while a resistance to third-generation cephalosporins was noted at, overall, low levels (1.8% and 1.2% for cefotaxime and ceftazidime, respectively) [35]. However, surveillance systems should be on alert to identify the differences in the resistance trends of the *Salmonella* strains.

The fluoroquinolone resistance in *Salmonella* is mainly caused by chromosomal mutations in the quinolone resistance-determining regions (QRDRs) of the topoisomerase genes *gyr*A, *gyr*B, *par*C and *par*E. In addition to the QRDR topoisomerase mutations, several plasmid-mediated quinolone resistance (PMQR) mechanisms have been described. The PMQR mechanisms, which are clinically important, result in a reduced susceptibility to ciprofloxacin but only to a modest or no increase in the susceptibility to nalidixic acid. With ciprofloxacin, an overlap in the inhibition zone diameters between wild-type isolates and isolates with low-level resistance has been reported for the 5-μg disk [36]. However, the pefloxacin 5-µg disk can be used to detect all the currently defined fluoroquinolone resistance mechanisms of *Salmonella* spp. According to our results, for the period when nalidixic acid and ciprofloxacin antibiotic disks were used, only 0.6% of the nalidixic resistant isolates exhibited a resistance to ciprofloxacin; thus, a low-level resistance and, possibly, PMQR-mediated-resistant isolates were not identified. When ciprofloxacin was replaced by pefloxacin, it seems that all fluoroquinolone-resistant isolates were identified, independently of the resistance mechanism, since all nalidixic-resistant isolates were also resistant to pefloxacin. The isolates only resistant to pefloxacin were determined, indicating, possibly, a PMQR mechanism.

## 4. Materials and Methods

### 4.1. Human Isolates

Human salmonellosis is one of the mandatory notifiable diseases in Greece, and the NPHO is notified about all diagnosed cases. In parallel, human *Salmonella* isolates, along with a short reporting form, including the patient’s demographics; date of sampling and specimen type (stool, blood, cerebrospinal fluid, pus or urine) are sent to the National Reference Centre for *Salmonella* (NRCS). The laboratory surveillance system is voluntary, and all the isolates routinely undergo serotyping and antimicrobial susceptibility testing. Molecular typing is performed in outbreak investigations and in the context of certain epidemiological studies.

The data for clinical *Salmonella* isolates was extracted directly from the database of the NRCS for the period from 2003 to 2020.

### 4.2. Strain Characterization of Clinical Isolates and Data Analysis

The serotyping of *Salmonella* spp. isolates was performed by the slide agglutination method according to the White-Kaufmann–Le Minor Scheme [37]. To confirm that strains serotyped as *S*. serovar 1,4,[5]:i:- were *S*. Typhimurium monophasic variants, one multiplex PCR assay was applied as described by Tennant et al. [16]. Between 2003 and 2013, susceptibility testing was performed by the agar disk diffusion method (Kirby-Bauer) according to the protocols of the Clinical and Laboratory Standard Institute (CLSI) [38]. Since 2014, susceptibility testing has been performed according to the European Committee on Antimicrobial Susceptibility Testing (EUCAST) [39]. Throughout the studied period, 2003–2020, several antibiotics were added or removed to meet certain requirements raised by the epidemiological data on the antibiotic resistance of *Salmonella* spp. In 2017, ciprofloxacin was replaced by pefloxacin in our panel. The reason for this change was that the nalidixic acid disk used as a screening test for resistance to fluoroquinolones does not adequately detect isolates with plasmid-mediated quinolone resistance (PMQR), and an overlap in the inhibition zone diameters between wild-type isolates and isolates with low-level resistance has been reported for the 5-μg ciprofloxacin disk. The replacement of the ciprofloxacin disk with that of pefloxacin 5μg in the disk diffusion method was in accordance with the guidelines of the ECDC [40]. Furthermore, azithromycin was added in 2020 to our panel. This specific antibiotic was added as one of the priority antimicrobials to be tested for *Salmonella* spp., since it is considered as the last resort drug for invasive salmonellosis [40].

The following antibiotics were tested: (i) penicillins: ampicillin and amoxicillin-clavulanic acid; (ii) cephalosporins: ceftazidime, ceftriaxone and cefotaxime; (iii) fluoroquinolones: ciprofloxacin, nalidixic acid and pefloxacin; (iv) miscellaneous agents: chloramphenicol, trimethoprim, sulfamethoxazole-trimethoprim and spectinomycin; (v) aminoglycosides: kanamycin, tobramycin, netilmicin and streptomycin; (vi) carbapenems: meropenem; (vii) macrolides: azithromycin and, finally, (viii) tetracyclines: tetracycline.

### 4.3. Statistical Analysis

The (para)typhoidal isolates were excluded from further analysis. The monthly counts of the NTS isolates were regressed against time from January 2003 to December 2020 using a negative binomial regression model to assess the temporal trend of the *Salmonella* isolation rate in the population. A negative binomial regression model was also fitted for assessing the seasonal variations. The number of serotypes identified and the proportion of each isolate over the total number of isolates were calculated. The annual temporal distribution of the isolation rate was graphed for the most frequently identified serotypes. The distribution of each serotype by the age and sex of the patients, as well as by the type of sample, was calculated. The proportion of antimicrobial-resistant isolates by serotype was estimated. The statistical analysis was conducted using Stata 16 statistical software (StataCorp. 2019. Stata Statistical Software: Release 16. College Station, TX: StataCorp LLC).

## 5. Conclusions

The laboratory surveillance data on human non-typhoidal *Salmonella* spp. is important in terms of providing early warnings for public health interventions and evaluating the impacts of certain veterinary measures against *Salmonella* spp. on humans. The globalization of the food industry and supply raises concerns about the global spread of foodborne diseases that can be best addressed through strong surveillance systems.

The whole-genome sequence-based surveillance of *Salmonella* spp. has been adopted by several countries, providing valuable information and enabling a vast amount of information and the highest possible resolution for pathogen subtyping. However, the implementation of whole-genome sequence-based typing methods for *Salmonella* spp. isolates during routine surveillance varies among the EU countries. Greece should invest, at the national level, in providing the required technical resources and expertise in order to strengthen public health surveillance. The conventional serotyping and antimicrobial test methods are valuable and should not be abandoned yet.

## Figures and Tables

**Table 1:** The number (*N*) of isolates and proportion (%) of the most frequently identified serotypes, Greece, 2003–2020.

Serotype *	*Ν*	%	Serotype	*Ν*	%
*S*. Enteritidis	5466	52.8%	*S*. Glostrup	35	0.3%
*S*. Typhimurium	1188	11.5%	*S*. Bispebjerg	33	0.3%
Monophasic *S*. Typhimurium	441	4.3%	*S*. Halle	32	0.3%
*S*. Bovismorbificans	350	3.4%	*S*. Mbandaka	31	0.3%
*S. enterica* subsp. *salamae* (II)	252	2.4%	*S*. Richmond	31	0.3%
*S*. Oranienburg	247	2.4%	*S*. Livingstone	28	0.3%
*S*. Kottbus	141	1.4%	*S*. Agona	27	0.3%
*S*. Blockley	140	1.4%	*S*. Derby	27	0.3%
*S*. Infantis	107	1.0%	*S*. Heidelberg	25	0.2%
*S*. Newport	106	1.0%	*S*. Montevideo	25	0.2%
*S*. Hadar	102	1.0%	*S*. Vari	25	0.2%
*S*. Muenchen	96	0.9%	*S*. Anatum	24	0.2%
*S*. Muenster	82	0.8%	*S*. Stanley	23	0.2%
*S*. Thompson	82	0.8%	*S*. Brandenburg	21	0.2%
*S*. Abony	77	0.7%	*S*. Coeln	21	0.2%
*S*. Kambole	63	0.6%	*S*. Miami	21	0.2%
*S*. Stanleyville	62	0.6%	*S*. Saintpaul	20	0.2%
*S*. Braenderup	54	0.5%	*S*. Cerro	19	0.2%
*S*. Senftenberg	47	0.5%	*S*. Give	19	0.2%
*S*. Virchow	47	0.5%	*S*. Chester	17	0.2%
*S*. Bredeney	45	0.4%	*S*. Szentes	17	0.2%
*S*. Corvallis	42	0.4%	*S*. Umbilo	17	0.2%
*S. enterica* subsp. *diarizonae* (IIIb)	39	0.4%	*S*. Meleagridis	16	0.2%
*S*. Bardo	36	0.3%			

* One hundred and forty-six *Salmonella* serotypes represented by less than 16 isolates each (< 0.2% of the total number of serotyped isolates) during the 18 years of the study period are not included in the table.

**Table 2:** Distribution of the *Salmonella* spp. serotypes by age groups of the persons whose specimens yielded isolates, 2003–2020, Greece (*N* = number, % = percentage).

Age Group	*S*. Enteritidis		*S*. Typhimurium		Monophasic *S*. Typhimurium		*S*. Bovismorbificans		*S*. Oranienburg		(*S*. II) 1,4,[5],12,[27]:b:-		Other Serotypes		Total (%)
	*N*	%	*N*	%	*N*	%	*N*	%	*N*	%	*N*	%	*N*	%	
0–11 months	167	27.5	74	12.2	16	2.6	43	7.0	28	4.6	26	4.4	254	41.8	608 (8.6%)
1–5 years	1324	55.7	336	14.1	132	5.5	56	2.4	60	2.5	46	1.9	427	17.9	2381 (33.8%)
6–14 years	903	64.4	148	10.6	86	6.1	24	1.7	20	1.3	11	0.8	211	15.0	1403 (19.9%)
15–64 years	1121	58.0	176	9.1	63	3.3	71	3.7	42	2.1	14	0.7	445	23.0	1932 (27.5%)
>= 65 years	326	45.8	74	10.4	17	2.4	41	5.8	26	3.7	5	0.8	223	36.7	712 (10.1%)

**Table 3:** The type of specimen where *Salmonella* spp. was isolated for *S*. Enteritidis, S Typhimurium, Monophasic *S*. Typhimurium, *S*. Bovismorbificans, *S*. Oranienburg, *S. enterica* subsp. *salamae* and all other serotypes, Greece, 2003–2020 (*N* = number, % = percentage).

Specimen	*S*. Bovismorbificans		*S*. Enteritidis		Monophasic *S*. Typhimurium		*S*. Oranienburg		*S*. Typhimurium		*Salmonella enterica* subsp. *salamae*		Other Serotypes		Total	
	*N*	%	*N*	%	*N*	%	*N*	%	*N*	%	*N*	%	*N*	%	*N*	%
Blood	16	4.8	204	3.8	7	1.7	28	12.5	56	4.9	6	2.8	112	5.4	429	4.4%
Cerebrospinal fluid	0	0.0	1	0.0	0	0.0	0	0.0	0	0.0	0	0.0	2	0.1	3	0.0%
Stool	305	92.4	5,040	95.1	411	97.4	191	85.3	1059	93.5	197	92.9	1881	91.0	9084	93.8%
Urine	6	1.8	29	0.5	3	0.7	2	0.9	7	0.6	8	3.8	48	2.3	103	1.1%
Pus	0	0.0	7	0.1	0	0.0	0	0.0	7	0.6	0	0.0	5	0.2	19	0.2%
Other	3	0.9	18	0.3	1	0.2	3	1.3	4	0.4	1	0.5	19	0.9	49	0.5%
Total	330	100.0	5299	100.0	422	100.0	224	100.0	1133	100.0	212	100.0	2067	100.0	9687	100.0%

**Table 4:** The antimicrobial resistance of *Salmonella* spp. isolates of the selected serovars to the selected antimicrobials, Greece, 2003–2020.

	Number of Isolates Tested for AMR	Penicillins	Cephalosporins	Carbapenems	Fluoroquinolones	Aminoglycosides	Macrolides	Tetracyclines	Miscellaneous Agents *
*S*. Bovismorbificans	279	6	2		2	31		2	41
%		2.2%	0.7%	0.00%	0.7%	11.1%	0.00%	0.7%	14.7%
*S*. Enteritidis	4720	131	62		845	64	1	49	200
%		2.8%	1.3%	0.00%	17.9%	1.4%	0.02%	1.0%	4.4%
Monophasic *S*. Typhimurium	419	336	6		14	336		358	283
%		80.2%	1.4%	0.00%	3.4%	80.2%	0.00%	85.4%	67.5%
*S*. Oranienburg	179	3	3			9	1	3	10
%		1.7%	1.7%	0.00%	0.0%	5.0%	0.56%	1.7%	5.6%
*S*. *enterica* subsp. *salamae*	204	8	9			50		5	17
%		3.9%	4.4%	0.00%	0.0%	24.5%	0.00%	2.5%	8.3%
*S*. Typhimurium	976	259	20		66	345	1	363	267
%		26.5%	2.0%	0.00%	6.7%	35.4%	0.10%	37.2%	27.3%
Other	1926	185	64		239	475	1	291	305
%		9.6%	3.3%	0.00%	12.4%	24.7%	0.05%	15.1%	15.8%
Total	8703	928	166		1166	1310	4	1071	1123
%		10.7%	1.9%	0.00%	13.0%	15.0%	0.05%	12.3%	12.0%

* Includes: sulphonamides, trimethoprim, chloramphenicol and streptomycin.

## Data Availability

The data presented in this study are available on request from the corresponding author.
